# Functional and Aesthetic Outcomes of the Subtarsal Approach in Zygomaticomaxillary Complex Fractures With Orbital Floor Involvement: A Prospective Cohort Study

**DOI:** 10.7759/cureus.93604

**Published:** 2025-09-30

**Authors:** Shahid Farooq, Mohd Younis Bhat, Reshu Rastogi, Anupam Bansal, Angel Angel, Reshma Hammannavar, Viraj Chaudhari

**Affiliations:** 1 Department of Dentistry, Government Medical College, Kathua, IND; 2 Department of Oral and Maxillofacial Surgery, Government Dental College and Hospital, Srinagar, IND; 3 Department of Dentistry, Rajendra Institute of Medical Sciences, Ranchi, IND; 4 Department of Oral and Maxillofacial Surgery, Darshan Dental College and Hospital, Udaipur, IND; 5 Department of Pedodontics and Preventive Dentistry, Desh Bhagat Dental College and Hospital, Mandi Gobindgarh, IND; 6 Department of Oral and Maxillofacial Surgery, Jawahar Medical Foundation's Annasaheb Chudaman Patil Memorial Dental College, Dhule, IND

**Keywords:** approach, orbital fractures, scarring, surgical, zygomatic fractures

## Abstract

Introduction

Zygomaticomaxillary complex (ZMC) fractures, often resulting from high-impact trauma, frequently involve the orbital floor, leading to functional and aesthetic complications such as diplopia, enophthalmos, and facial asymmetry. The subtarsal approach, which involves an incision through the skin, provides access to the orbital floor while potentially reducing complications. This study aimed to evaluate the efficacy and safety of the subtarsal approach in managing ZMC fractures with orbital floor discontinuity, focusing on functional outcomes (nerve paresthesia, ocular movement, and visual acuity), lower eyelid position, fracture stability, and scar visibility at one and three months post-surgery.

Materials and methods

A prospective cohort study was conducted in the Department of Oral and Maxillofacial Surgery at Annasaheb Chudaman Patil Memorial Dental College, Dhule, Maharashtra, between February 2022 and February 2024. Twenty-four patients aged 18-65 years with displaced ZMC fractures and orbital floor discontinuity confirmed via CT were included. The exclusion criteria were prior orbital surgery, severe comorbidities, ocular pathology, infected or comminuted fractures, and refusal to consent. Preoperative assessments included clinical examinations of facial symmetry, ocular motility, visual acuity, and nerve function, along with CT imaging. The subtarsal approach involved a 4-7 mm incision below the lash line, with layered dissection to access the orbital floor for fracture reduction and fixation using titanium miniplates and mesh. Outcomes were assessed at one and three months for nerve paresthesia, eyelid position, ocular movement, visual acuity, fracture stability, and scar visibility using the Patient and Observer Scar Assessment Scale (POSAS). Statistical analysis employed chi-square and Wilcoxon signed-rank tests, with p < 0.05 indicating significance.

Results

The study population was predominantly male, with tetrapod fractures being the most common type. All patients exhibited periorbital edema, 18 (75%) had infraorbital nerve dysfunction, and 14 (58.3%) had reduced visual acuity at 0.3 preoperatively. By three months post-surgery, significant improvements were observed: nerve paresthesia decreased (p = 0.017), normal eyelid position increased (p = 0.009), and visual acuity improved (p = 0.012). Fracture stability scores improved from a median of 5 (mean 5.17 ± 0.702) to 1 (mean 1.21 ± 0.833, p = 0.001), and scar evaluation scores decreased from a median of 7 (7.33 ± 1.007) to 3 (3.33 ± 0.637, p = 0.001). The improvement in ocular movement from full restriction was not statistically significant (p = 0.08).

Conclusion

The subtarsal approach proved effective and safe, achieving significant functional and aesthetic improvements in ZMC fracture management with minimal complications. Further studies with larger sample sizes and longer follow-up periods are required to validate these findings.

## Introduction

The zygomaticomaxillary complex (ZMC) is a critical anatomical structure of the midface that contributes to cheek prominence, orbital integrity, and overall facial aesthetics and function [1]. ZMC fractures are among the most common maxillofacial injuries and typically result from high-impact trauma, such as motor vehicle accidents or assaults, owing to the prominent anatomical projection of the zygoma [1,2]. These fractures often involve disruption of the zygomatic bone and maxillary buttresses and frequently extend to the orbital floor, leading to potential complications, including diplopia, enophthalmos, and restricted ocular motility, if not adequately addressed [3,4]. Effective surgical intervention requires precise reduction and stable fixation to restore form and function, given the complex relationship between the zygoma and surrounding structures [5].

Treatment strategies for ZMC fractures range from conservative management for minimally displaced fractures to surgical intervention for complex cases [5,6]. Open reduction and internal fixation (ORIF) using miniplates remains the gold standard, with coronal, buccal sulcus, and transconjunctival approaches being commonly employed [6]. Traditional approaches, such as transconjunctival or subciliary incisions, have been used to minimize visible scarring and preserve eyelid function; however, each carries inherent risks of postoperative complications, such as ectropion or scleral show [7].

In this study, the efficacy and outcomes of the subtarsal approach, as a refined transcutaneous method to access the orbital floor in patients with ZMC fractures, were evaluated. The subtarsal approach involves a horizontal incision 4-7 mm below the lower eyelid lash line, aligned with a natural crease, followed by dissection through the skin and orbicularis oculi muscle to access the orbital floor. It provides good exposure for fracture repair or reconstruction while minimizing eyelid malposition and scarring [4,8,9].

Palavalli MH et al. [9] conducted a systematic review to evaluate the efficacy of various surgical procedures for orbital fractures and concluded that the transconjunctival approach was associated with the highest rate of complications (36.19%), while the subtarsal approach had the lowest rate (8.2%). Another systematic review reported that the transconjunctival technique produced markedly superior aesthetic results compared with the subtarsal technique. Nevertheless, the subtarsal technique is associated with a lower frequency of postoperative complications, including hyperesthesia, entropion, ectropion, enophthalmos, and epiphora [10].

This study aimed to evaluate the efficacy and safety of the subtarsal approach for accessing the orbital floor in the surgical management of ZMC fractures, with a focus on functional and aesthetic outcomes. The objectives were to assess postoperative nerve paresthesia (infraorbital nerve function), lower eyelid position (to detect ectropion or scleral show), ocular movement (to evaluate diplopia or restriction), visual acuity, fracture stability, and scar visibility at one and three months post-surgery.

## Materials and methods

This prospective cohort study was conducted in the Department of Oral and Maxillofacial Surgery, Jawahar Medical Foundation's Annasaheb Chudaman Patil Memorial Dental College, Dhule, Maharashtra, between February 2022 and February 2024. The study protocol was approved by the Institutional Ethics Committee (EC/NEW/INST/2022/2959/SS23), and written informed consent was obtained from all enrolled patients prior to inclusion. The study adhered to the principles of the Declaration of Helsinki.

Patient eligibility

Patients aged 18-65 years who presented with unilateral or bilateral ZMC fractures and orbital floor discontinuity confirmed by CT were included. The inclusion criteria were displaced ZMC fractures (Class III to V according to Rowe and Killey’s classification) [11] requiring ORIF and orbital floor defects necessitating reconstruction. Exclusion criteria included previous orbital or zygomatic surgery, severe comorbidities (ASA Class III or higher), ocular pathology, infected fractures of the orbito-zygomatico-maxillary complex region, comminuted ZMC fractures (Classes VI and VII), or refusal to consent.

Sample size estimation was performed using G*Power software with a power of 80% and an alpha level of 5%. An effect size of 0.52, derived from a reference study on scar healing scores at multiple time points in the infraorbital region, was applied [12]. Based on these inputs, the calculated total sample size for this study was 24 patients, ensuring sufficient power to detect statistically significant differences in scar healing outcomes over time.

Patients with suspected ZMC fractures underwent comprehensive preoperative evaluation. Radiological examinations were conducted for all selected subjects using conventional radiographs, including submentovertex and paranasal sinus views, and/or advanced imaging modalities such as CT of the facial bones, to confirm the diagnosis and assess the extent of ZMC fractures with orbital floor discontinuity. Additionally, routine hematological investigations and serological tests were performed for all patients to evaluate overall health status. Subjects were screened to ensure fitness for surgery under general anesthesia, adhering to standard preoperative protocols.

Detailed medical histories, including mechanism of injury, associated trauma, and comorbidities, were collected. Clinical examinations assessed facial symmetry, ocular motility, diplopia, and enophthalmos (measured using a calibrated Hertel exophthalmometer; Oculus Inc., Wetzlar, Germany), along with sensory deficits along the infraorbital nerve. Visual acuity and globe integrity were evaluated by an ophthalmologist to rule out ocular pathology.

**Figure 1:**
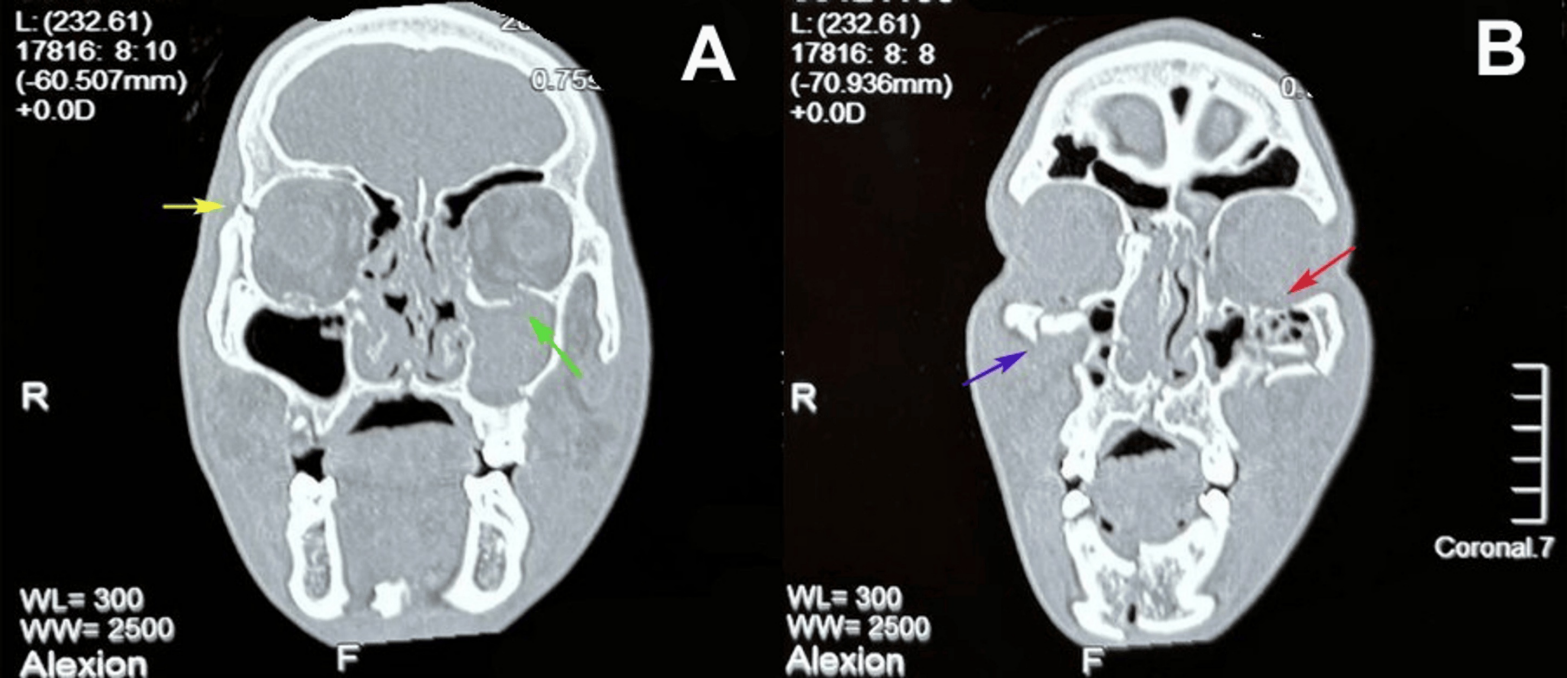
Evaluation of zygomaticomaxillary complex (ZMC) fracture with orbital floor involvement using CT. (A) Coronal CT scan showing a fracture of the lateral wall of the right orbit (yellow arrow) and left orbital floor discontinuity (green arrow), demonstrating disruption of the infraorbital rim and orbital floor. (B) Sagittal CT scan illustrating the extent of the orbital floor defect (red arrow) and zygomatic displacement (blue arrow). Original CT images of a patient from the study, used with the patient’s permission.

Radiographic assessment was performed using a high-resolution CT scanner (Somatom Definition Edge, Siemens Healthineers, Erlangen, Germany) to confirm ZMC fractures with orbital floor discontinuity and classify fracture displacement (Figure 1). Scans were acquired in the axial, coronal, and sagittal planes with a slice thickness of 1 mm, a field of view of 200 mm, a bone reconstruction algorithm (B70s kernel), a tube voltage of 120 kVp, a tube current of 200 mA, and a pitch of 0.8.

All clinical assessment tools were calibrated prior to use to ensure measurement accuracy. Before each session, the Hertel exophthalmometer (Oculus Inc., Wetzlar, Germany) was calibrated according to the manufacturer’s guidelines to verify alignment and precision in measuring enophthalmos. For clinical assessments, including enophthalmos, scar evaluation using the Patient and Observer Scar Assessment Scale (POSAS) [13], and sensory testing along the infraorbital nerve distribution, reliability was evaluated to ensure consistency. Inter-observer reliability was assessed by two independent examiners who performed measurements on a subset of 10 patients during the same visit. The intraclass correlation coefficient (ICC) was calculated, with values >0.75 considered acceptable. For the POSAS, both the patient and observer components (vascularity, pigmentation, thickness, relief, pliability, pain, and itching) were evaluated for agreement. Sensory testing for paresthesia was standardized using a two-point discrimination test and light touch assessment with a cotton wisp.

Following nasoendotracheal or oral intubation, the surgical field was aseptically prepared using a 5% povidone-iodine solution (Betadine; Purdue Pharma, Stamford, CT, USA) and draped according to the standard protocol. A forced duction test was performed to assess extraocular muscle entrapment and restrictions on globe mobility. A corneal shield (EyeGard, Bausch & Lomb, Rochester, NY, USA) with lubricating solution was placed to protect the eye during surgery. A horizontal incision was made 4-7 mm below the lower eyelid lash line, aligned with a natural skin crease. Local infiltration was performed with 2% lignocaine containing 1:200,000 epinephrine (Xylocaine; AstraZeneca, Cambridge, UK) in the lower eyelid and infraorbital regions to achieve vasoconstriction and reduce intraoperative bleeding.

**Figure 2:**
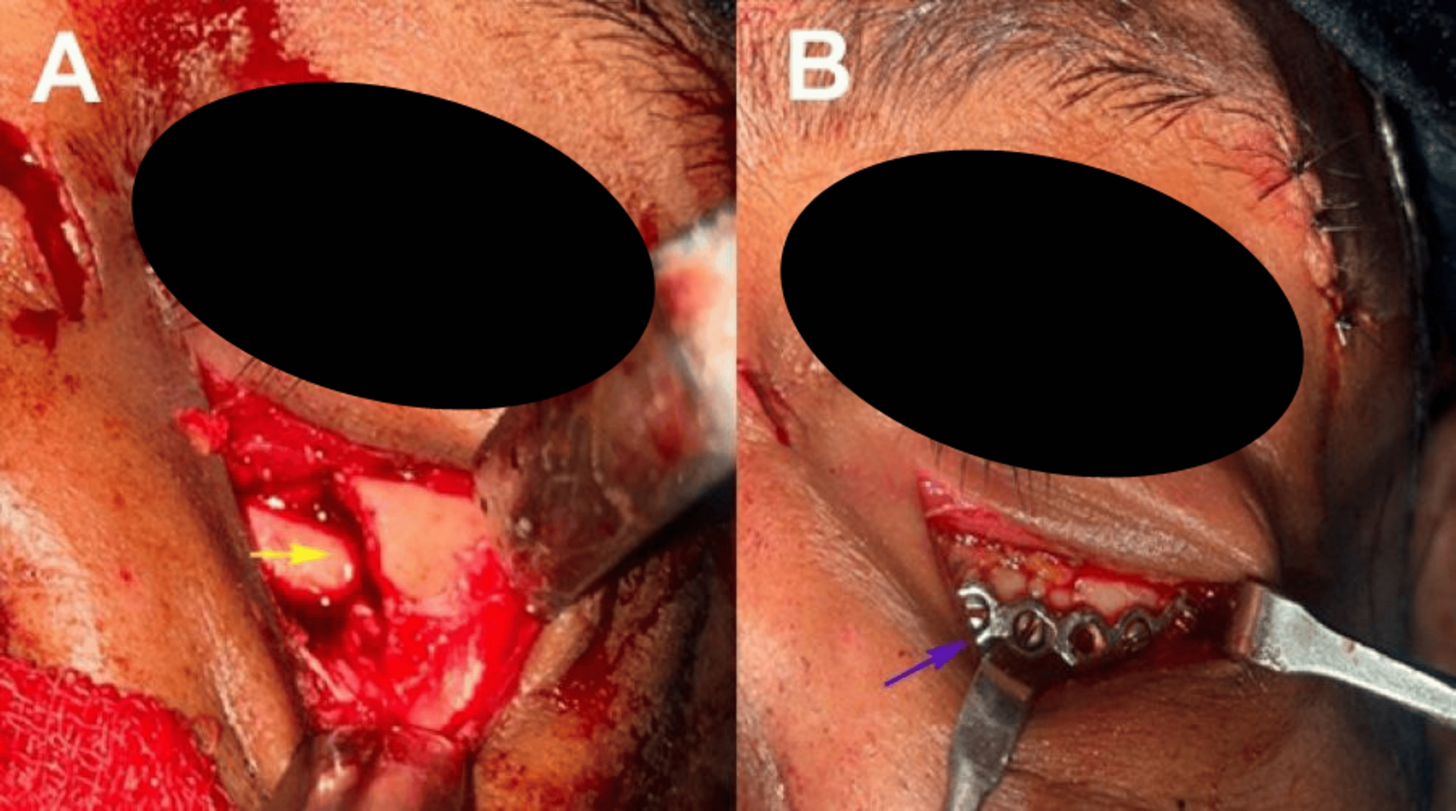
Surgical exploration using the subtarsal approach for left zygomaticomaxillary complex (ZMC) fracture repair (A) Intraoperative view showing the subtarsal incision (4–7 mm below the lower eyelid lash line) and dissection through the orbicularis oculi muscle to expose the orbital septum and infraorbital rim (yellow arrow), (B) Placement of a contoured 1.5 mm titanium miniplates (blue arrow) for orbital floor reconstruction and fracture fixation, ensuring anatomical reduction and support of orbital contents. Images are from a study patient, used with permission.

The lower eyelid was gently retracted and an incision was made through the skin and orbicularis oculi muscle using a No. 15 scalpel blade. Dissection proceeded through the muscle in line with the incision, exposing the orbital septum. The septum was incised to access the periosteum of the infraorbital rim, followed by subperiosteal dissection to expose the infraorbital rim, orbital floor, and lateral orbital wall. This provided wide exposure for fracture reduction and reconstruction while preserving the integrity of the pretarsal orbicularis muscle. A maxillary vestibular incision was made from the canine to the first molar to access and reduce the zygomatic buttress fracture. The fracture was anatomically reduced along both vertical and horizontal planes at the frontozygomatic suture, infraorbital rim, and zygomatic buttress. Entrapped orbital fat or muscle was released and repositioned within the orbit. Fixation was achieved using 1.5 mm titanium miniplates and screws (MatrixNEURO, DePuy Synthes, Raynham, MA, USA) and a contoured titanium mesh (Orbital Floor Mesh, DePuy Synthes, Raynham, MA, USA) to support orbital contents. A final forced duction test was performed to ensure decompression (Figure 2).

In cases involving concurrent mandibular or maxillary fractures, appropriate reduction and fixation were performed using 2 mm miniplates and screws (MatrixMandible, DePuy Synthes, Raynham, MA, USA) under maxillomandibular fixation (MMF). Hemostasis was achieved, and the surgical field was irrigated with antiseptic (Betadine) and saline solutions. Closure was performed in the following layers: orbicularis oculi with 4-0 polyglactin 910 (Vicryl, Ethicon Inc., Somerville, NJ, USA), orbital septum with 5-0 polyglactin 910 (Vicryl), intraoral mucosa with 4-0 polyglactin 910 (Vicryl), and skin with 5-0 polypropylene (Prolene, Ethicon Inc., Somerville, NJ, USA) in a running or interrupted manner. The sclera was irrigated with saline, and neomycin ophthalmic ointment (Neosporin; GlaxoSmithKline, Brentford, UK) was applied with eye padding.

All patients received intravenous antibiotics, including amoxicillin-clavulanic acid (Augmentin, 1.2 g twice daily; GlaxoSmithKline, Brentford, UK) and metronidazole (Flagyl, 500 mg thrice daily; Pfizer, NY, USA), for five days to prevent postoperative infection, in accordance with institutional protocols for maxillofacial surgery. Pain was managed with paracetamol (Crocin, 1 g twice daily; GlaxoSmithKline, Brentford, UK) for five days. Patients were advised to avoid strenuous activities and maintain a soft diet for two weeks.

Outcome assessment

Patients were followed up at one month and three months postoperatively for clinical and radiographic evaluations. Clinical evaluation was performed at each follow-up visit to assess multiple outcomes. Nerve paresthesia was evaluated using standardized sensory testing along the infraorbital nerve distribution, with two-point discrimination and light-touch assessment. The position of the lower eyelid was assessed for ectropion or scleral show during clinical examination and photographic documentation. Ocular movement was evaluated for diplopia or restriction using a duction test and patient-reported symptoms. Visual acuity was measured using Snellen charts by an ophthalmologist. Fracture stability was confirmed by clinical palpation and radiographic imaging of alignment and implant integrity. Scar visibility was assessed using the POSAS at one and three months, with parameters evaluated under standardized conditions. Scar vascularity was examined through visual inspection under uniform lighting and the blanching technique, in which light pressure was applied using a finger or glass slide to assess capillary refill and reperfusion. Postoperative radiological investigations were performed at three months using the same parameters as the preoperative studies to assess fracture reduction and implant stability. Two observers (Shahid Farooq and Mohd Younis Bhat) independently evaluated the scans, and inter-observer reliability was assessed using the ICC to ensure consistency in radiographic findings.

Statistical analysis

Data analyses were performed by a statistician (Angel Angel) using coded data. The SPSS software (version 27.0; IBM Corp., Armonk, NY, USA) was used for analyses. Normality was assessed using the Kolmogorov-Smirnov test, which revealed a non-normal distribution and necessitated the use of non-parametric tests. Continuous variables are presented as mean, median, and standard deviation, whereas categorical variables are summarized as frequencies and percentages. Associations between categorical variables were evaluated using the chi-square test. The Wilcoxon signed-rank test was employed to compare scar and fracture stability scores at multiple time points. Statistical significance was set at p < 0.05.

## Results

The baseline characteristics of the study sample revealed a predominance of males compared to females. Regarding the types of ZMC fractures, classic tetrapod/tripod fractures involving all four components were the most common, followed by isolated lateral orbital wall, inferior orbital rim, and arch fractures. All patients exhibited periorbital edema. Visual acuity ranged from 0.3 in 14 (58.33%) patients to 0.5 in 10 (41.67%) patients. Infraorbital nerve dysfunction was present in 18 (75%) patients. Lower eyelid malposition was equally distributed between the ectropion and retraction groups. Bilateral orbital floor involvement was observed in 13 (54.17%) patients. These findings highlight the varied presentations of ZMC fractures, with significant functional and anatomical implications (Table 1).

**Table 1:** Baseline characteristics of patients with zygomaticomaxillary complex (ZMC) fractures with orbital floor involvement treated using the subtarsal approach. Data are presented as frequency (N) and percentage (%), where n denotes the number of patients. All fracture types had confirmed orbital floor discontinuity on computed tomographic imaging. Percentages may not sum to 100% due to rounding.

Parameters	Category	N	Percentage
Sex	Females	9	37.5
	Males	15	62.5
Type of ZMC fracture	Isolated arch fracture	3	16.67
	Isolated lateral orbital wall fracture	6	25
	Isolated inferior orbital rim fracture	5	20.83
	Fracture of all four (classic tetrapod/tripod)	9	37.5
Periorbital edema	Yes	24	100
Visual acuity (decimal)	0.3	14	58.33
	0.5	10	41.67
Infraorbital nerve dysfunction	No	6	25
	Yes	18	75
Lower eyelid position	Ectropion	12	50
	Retraction	12	50
Bilateral orbital floor fracture	No	11	45.83
	Yes	13	54.17

Analysis of independent associations at different time points using the chi-square test revealed significant improvements in several parameters. Nerve paresthesia was significantly reduced from 9 (37.5%) patients at one month to 3 (12.5%) patients at three months (p = 0.017). Similarly, the proportion of patients with normal lower eyelid position increased significantly from one month to three months (p = 0.009), while ectropion cases declined. Visual acuity also improved significantly, with normal vision increasing from one to three months (p = 0.012). Although ocular movement showed improvement between the two time points, the change was not statistically significant (p = 0.08). Overall, these results indicate significant functional recovery in terms of nerve sensation, eyelid position, and visual acuity within three months postoperatively (Table 2).

**Table 2:** Analysis of postoperative outcomes of the subtarsal approach for zygomaticomaxillary complex (ZMC) fractures at one and three months. Data are presented as frequency (N) and percentage (%) for categorical postoperative outcomes at one and three months, where n denotes the number of patients. *p < 0.05 denotes statistical significance using the chi-square test.

Parameter	Category	At one month N (%)	At three months N (%)	Test statistic	p-value
Nerve paresthesia	No	15 (62.50)	21 (87.50)	5.71	0.017*
	Yes	9 (37.50)	3 (12.50)		
Lower eyelid position	Normal	16 (66.67)	21 (87.50)	6.86	0.009*
	Ectropion	8 (33.33)	3 (12.50)		
Ocular movement	Full	8 (33.33)	19 (79.17)	3.16	0.08
	Partial	16 (66.67)	5 (20.83)		
Visual acuity (decimal)	1	10 (41.67)	18 (75.00)	5.78	0.012*
	0.5	9 (37.50)	6 (25.00)		
	0.3	5 (20.83)	0 (0.00)		

Comparative analysis using the Wilcoxon signed-rank test revealed significant improvement in both fracture stability and scar evaluation scores between one month and three months. The median fracture stability score decreased significantly from 5 to 1 (mean ± SD: 5.17 ± 0.702 to 1.21 ± 0.833, p = 0.001). Similarly, scar evaluation scores improved significantly from a median of 7 to 3 (mean ± SD: 7.33 ± 1.007 to 3.33 ± 0.637, p = 0.001). These results indicate a marked and statistically significant healing progression over time (Table 3).

**Table 3:** Comparison of fracture stability and scar evaluation outcomes of the subtarsal approach for zygomaticomaxillary complex (ZMC) fractures at one and three months. Data are presented as median, mean, and SD for continuous/ordinal postoperative outcomes at one and three months. *p = 0.001 denotes statistical significance using the Wilcoxon signed-rank test. Fracture stability was scored on a 0-6 scale (0 = fully stable, 6 = severe instability) based on radiographic and clinical assessment. Scar evaluation was performed using the Patient and Observer Scar Assessment Scale (POSAS), with total scores ranging from 6 to 60.

Parameter	Time point	Median	Mean	SD	Minimum	Maximum	z-value	p-value
Fracture stability	At one month	5	5.17	0.702	4	6	4.36	0.001*
	At three months	1	1.21	0.833	0	2		
Scar evaluation	At one month	7	7.33	1.007	5	9	4.34	0.001*
	At three months	3	3.33	0.637	2	4		

## Discussion

The subtarsal approach for managing ZMC fractures with significant orbital floor discontinuity represents a promising advancement in maxillofacial trauma care. The findings of this study highlight the potential of addressing a challenging subset of injuries, where achieving adequate exposure and optimal outcomes is critical. For the 24 patients in our cohort, each navigating the physical and emotional toll of facial trauma, the subtarsal approach offered a pathway to recovery marked by minimal complications, effective pain resolution, and aesthetically pleasing results. These outcomes resonate deeply, as they reflect not only clinical success but also the restoration of confidence and quality of life for individuals affected by such injuries.

Our results demonstrated that a combination of ZMC and orbital floor fractures occurred more frequently in males than in females, which aligns with a previous study [3]. Road traffic accidents were a major cause of such fractures. Our study revealed that classic tetrapod/tripod fractures were the most common, followed by lateral orbital wall fractures. Patil AJ et al. [14] similarly identified road traffic accidents as the principal contributor in 11 (64.7%) patients, followed by falls from elevation in 3 (17.6%) patients. It was also noted that the most common site of fracture was the lateral orbital wall.

The significant improvements observed in postoperative outcomes underscore the efficacy of the subtarsal approach. The reduction in nerve paresthesia from 37.5% at one month to 12.5% at three months indicates robust recovery of infraorbital nerve function, likely due to meticulous surgical techniques that minimize nerve traction during dissection [10,12,15]. Similarly, the increase in normal lower eyelid position and decline in ectropion reflect the advantage of the subtarsal approach in preserving the integrity of the pretarsal orbicularis oculi muscle and reducing lid malposition compared to the traditional subciliary approach [16]. The significant improvement in visual acuity suggests effective orbital floor reconstruction, restoring orbital volume and globe position, thereby alleviating functional deficits such as diplopia or enophthalmos [9]. However, the non-significant improvement in ocular movement may indicate persistent minor muscle entrapment or scarring in some patients, necessitating longer follow-up or adjunctive therapy.

Fracture stability and scar evaluation also demonstrated significant improvement, with the median fracture stability score dropping from 5 to 1 and the scar score improving from 7 to 3 by three months. These findings highlight the ability of the subtarsal approach to achieve stable anatomical reduction using titanium miniplates and meshes. The improved scar outcomes reflect the placement of the incision in a natural eyelid crease, minimizing visibility compared to the subciliary approach [10,16,17]. These results suggest that the subtarsal approach is reliable for balancing functional restoration and aesthetic outcomes in ZMC fracture repair.

Strobel L et al. [17] compared the subtarsal approach and transconjunctival approach in a cohort of 45 subjects who underwent orbital reconstruction procedures. The complication rates were similar across both surgical methodologies, with no statistically significant differences. Among the cohort subjected to the subtarsal procedure, discrete scar formation was recorded in seven of the 30 cases. However, no statistically significant variations were observed in terms of conspicuous scarring and asymmetries between the two surgical techniques, as evaluated by both non-expert and expert observers. The authors concluded that the subtarsal approach is safe and aesthetically advantageous for orbital reconstructions. Mahajan RK et al. [18] reported that the subtarsal incision approach has emerged as a beneficial technique for addressing infraorbital rim fractures and facilitating exploration of the orbital floor, yielding favorable results with respect to ectropion, scleral show, and scar formation.

Clinical implications

The subtarsal approach offers a favorable balance between wide surgical exposure and reduced complications, making it a preferred method for ZMC fractures with orbital floor involvement. The significant recovery of nerve function and visual acuity suggests that this approach effectively addresses functional deficits and improves patient quality of life. The choice of surgical incision must consider preoperative aesthetic, anatomical, and technical factors. For example, in cases where the patient exhibits downward gaze and laxity of the lower eyelid, there may be a higher risk of inferior eyelid malposition and subsequent ectropion. Therefore, incision selection should be individualized, based on both patient characteristics and surgeon preference.

Limitations

This study has several limitations. The small sample size may limit statistical power, particularly for detecting subtle differences in outcomes such as ocular movement. The preoperative presence of ectropion and retraction in 100% of patients is unusual and may reflect unaccounted preoperative trauma, requiring further validation. The three-month follow-up period may not capture long-term complications, such as delayed scar hypertrophy or persistent diplopia. Additionally, this technique has not yet been compared with alternative approaches and requires validation through multicenter randomized controlled trials with long-term follow-up.

## Conclusions

This study demonstrated that the subtarsal approach is an effective and reliable method for managing ZMC fractures with orbital floor discontinuity. It resulted in significant improvements in nerve function, lower eyelid position, visual acuity, fracture stability, and scar aesthetics within the three-month postoperative period. The ability of this approach to provide adequate surgical exposure while minimizing complications, such as ectropion and visible scarring, supports its clinical utility in achieving both functional restoration and favorable cosmetic outcomes. Despite these strengths, the study underscores the importance of careful patient selection and precise surgical technique to optimize results. Future research with larger sample sizes and longer follow-up periods is warranted to validate these findings and evaluate long-term outcomes.
